# Descriptive study of reported cases of sexual violence and specialized care services in the state of Minas Gerais, Brazil, 2019

**DOI:** 10.1590/S2237-96222023000300002.en

**Published:** 2023-10-23

**Authors:** Isabella Vitral Pinto, Iracy Silva Pimenta, Maria Bevilacqua Alves, Ana Pereira dos Santos, Cristiane Magalhães de Melo, Janete Gonçalves Evangelista, Kate Aparecida Rocha Lacerda, Paula Dias Bevilacqua

**Affiliations:** 1 Instituto René Rachou - Fundação Oswaldo Cruz , Grupo de Pesquisa Violências, Gênero e Saúde, Belo Horizonte, MG, Brasil; 2 Prefeitura de Belo Horizonte, Secretaria Municipal de Assistência Social, Segurança Alimentar e Cidadania, Belo Horizonte, MG, Brasil; 3 Universidade Federal de Viçosa, Programa de Pós-Graduação em Engenharia Agrícola – Recursos Hídricos e Ambientais, Viçosa, MG, Brasil; 4 Universidade Federal de Viçosa, Departamento de Serviço Social, Viçosa, MG, Brasil

**Keywords:** Gender-based Violence, Childhood Sexual Abuse, Abuse Notification, Health Information Systems, Descriptive Epidemiology, Violencia de Género, Abuso Sexual Infantil, Notificación Obligatoria, Sistemas de Información en Salud, Epidemiología Descriptiva, Violência de Gênero, Abuso Sexual na Infância, Notificação de Abuso, Sistemas de Informação em Saúde, Epidemiologia Descritiva

## Abstract

**Objective:**

to describe the sociodemographic profile of reported cases of sexual violence (SV) and the distribution of care services for this health condition in the state of Minas Gerais, Brazil, 2019.

**Methods:**

this was a descriptive study of the cases of sexual violence reported on the Notifiable Health Conditions Information System and care services registered in the National Health Establishment Registry.

**Results:**

a total of 4,418 notifications of SV were identified during the study period, representing more than 12 notifications per day; the majority of notifications were among females (87.0%) and among children and adolescents (72.0%); the distribution of care services showed care gaps in four of the 14 health macro-regions of the state of Minas Gerais; the maximum distances traveled to access referral services ranged from 93 to 327 km.

**Conclusion:**

the scarcity of care services for people subjected to sexual violence in the micro-regions and macro-regions of Minas Gerais highlights the need for planning public policies aimed at increasing access to these services.

## Study contributions

**Main results:**

Over 12 notifications of sexual violence were reported per day in the state of Minas Gerais in 2019, with a higher prevalence in females, children and adolescents, mixed-race/Black people. Care gaps were identified in four macro-regions of the state.

**Implications for services:**

There was a need for victims to travel long distances to receive care in municipalities with referral services for comprehensive care for sexual violence, which may hinder access and timely care.

**Perspectives:**

It is expected that the results can contribute to improving public policies, considering the need to strategically plan the location of specialized services for people subjected to sexual violence.

## RESUMEN

**Objetivo:**

describir el perfil sociodemográfico de los casos notificados de violencia sexual (VS) y la distribución de los servicios para esta condición.

**Métodos:**

estudio observacional descriptivo basado en datos del Sistema de Información de Enfermedades de Declaración Obligatoria y servicios registrados en el Registro Nacional de Estabelecimientos de Salud, en Minas Gerais, Brasil, en 2019.

**Resultados:**

se identificaron 4.418 notificaciones de SV que representan más de 12 notificaciones por día; los registros se centraron en el sexo femenino (87,0%), niños y adolescentes (72,0%); la distribución de los servicios mostró brechas de atención en cuatro macrorregiones entre las 14 existentes; la distancia máxima recorrida para asistencia en un servicio de referencia osciló entre 93 y 327 km.

**Conclusión:**

la escasez de servicios de atención a personas en situación de SV en las micro y macro regiones, indican la necesidad de planificación de políticas públicas para ampliar el acceso.

## RESUMO

**Objetivo:**

descrever o perfil sociodemográfico dos casos notificados de violência sexual (VS) e a distribuição dos serviços de atendimento ao agravo em Minas Gerais, Brasil, 2019.

**Métodos:**

estudo descritivo dos casos notificados no Sistema de Informação de Agravos de Notificação e dos serviços registrados no Cadastro Nacional dos Estabelecimentos de Saúde.

**Resultados:**

foram identificadas 4.418 notificações de VS no período, correspondendo a mais de 12 notificações/dia; houve maior frequência de notificações no sexo feminino (87,0%) e entre crianças e adolescentes (72,0%); a distribuição dos serviços mostrou vazios assistenciais em quatro das 14 macrorregiões de saúde do estado; as distâncias máximas percorridas para atendimento em serviços de referência variaram de 93 a 327 km.

**Conclusão:**

a escassez de serviços de atendimento a pessoas em situação de VS nas microrregiões e macrorregiões de Minas Gerais sinalizam a necessidade de planejamento de políticas públicas visando aumentar o acesso a esses serviços.

## INTRODUCTION

Sexual violence is defined as any forced, coerced, or threatened sexual act, including rape, attempted rape, unwanted sexual touching, or non-contact forms of sexual violence.^1^ Although it can occur among both women and men, gender inequality contributes to girls and women, Black race/skin color, transgender women and/or people with disabilities being more likely to experience sexual violence.^2^

Local social characteristics, such as (i) lower levels of education, (ii) attitudes and norms that normalize violence and gender inequalities, (iii) lack of employment opportunities for women, (iv) gender-discriminatory laws concerning property, marriage, divorce, (v) beliefs in family honor and sexual purity, (vi) ideologies of male sexual entitlement, and (vii) weak legal sanctions for sexual violence, pose a greater risk for the occurrence of this condition.^3^ Worldwide, one in three women has been subjected to physical and/or sexual violence by an intimate partner and/or non-partner at least once in their lifetime; and 13% have experienced such violence in the past 12 months.^3^

In Brazil, 66,348 cases of rape were reported in 2019, with 5,009 of them in the state of Minas Gerais, 85% and 86%, respectively, among females.^4^ It is common knowledge that female rape survivors face social stigmas, feelings of guilt for having experienced sexual violence, rejection from their families and communities, inducing factors that contribute to the underreporting of records of this form of violence^3^,^4^ and hinder its visibility. Therefore, the need for public policies aimed at addressing sexual violence is widely acknowledged.^5^,^6^

In addition to providing care for survivors of sexual violence, the health sector, together with Public Security, plays an important role in data collection on the phenomenon. Since 2011, cases of sexual violence have been compulsorily reported on the Notifiable Health Conditions Information System (*Sistema de Informação de Agravos de Notificação* - SINAN).^7^ Cases of sexual violence should be reported to the municipal health department within a 24hour period, upon gaining knowledge of the case.^8^ This urgency is justified by the need to ensure immediate access to prophylaxis for the human immunodeficiency virus (HIV) and other sexually transmitted infections (STIs), as well as emergency contraception for people who may get pregnant.^9^ In Brazil, care for people subjected to sexual violence is guaranteed by Law No. 12,845, of August 1, 2013, which ensures the provision of emergency, comprehensive and multidisciplinary care by hospitals.^10^ In order to organize the care network for these cases, Ordinances No. 485, of April 1, 2014, No. 618, of July 18, 2014, and No. 1,662, of October 2, 2015, redefined the operation of Care Service No. 165 for People Subjected to Sexual Violence (*Serviço nº 165 de Atenção às Pessoas em Situação de Violência Sexual* - SAP/VS) within the Brazilian National Health System (*Sistema Único de Saúde* - SUS), organized according to four classifications:^11-13^ Comprehensive Care for People Subjected to Sexual Violence (*Atenção Integral às Pessoas em Situação de Violência Sexual* - AIP/VS); Outpatient Care for People Subjected to Sexual Violence (*Atenção Ambulatorial às Pessoas em Situação de Violência Sexual* - AAP/VS); Termination of Pregnancy (*Interrupção da Gravidez* - IG), in the cases provided for by law; and Sexual Assault Evidence Collection (*Coleta de Vestígios de Violência Sexual* - CV/VS).

In Minas Gerais, a state with 853 municipalities, all four classifications of SAP/VS can be found within the SUS. However, despite the mandatory hospital care^10^ and the organization of SAP/VS,^11-13^ a survey conducted in 2017 showed that specialized services provided were insufficient to meet the demands for care, leading to care gaps.^14^

The aim of this study was to describe the sociodemographic profile of reported cases of sexual violence and the distribution of care services for this health condition.

## METHODS

This was a descriptive study of cases of sexual violence reported on the SINAN in Minas Gerais, in 2019, and the distribution of SAP/VS in the state of Minas Gerais, registered in the Brazilian National Health Establishment Registry (*Cadastro Nacional dos Estabelecimentos de Saúde* - CNES), in July 2019.

All notifications made in 2019 with the form of violence indicated as “sexual violence” in the Notification Form were included, regardless of sex or age group of the case.

The following variables were used according to sex (female; male) and age groups [in years: 0 to 9 (children); 10 to 19 (adolescents); 20 or over (adults)]:

a) demographic – race/skin color (White; Black; Asian; mixed-race; Indigenous; unknown) and marital status (single; married or in consensual union; widowed; separated; not applicable; unknown) –;
b) disability status (has a disability; does not have a disability; unknown);
c) pregnancy status (yes; no; not applicable; unknown);
d) previous occurrence of violence (yes; no; unknown);
e) place of occurrence (home; collective housing; school; sports facility; bar or similar establishment; public thoroughfare; trade/services; industries/construction site; other; unknown);
f) type of sexual violence (sexual harassment; rape; child pornography; sexual exploitation; others);
g) number of aggressors involved (one; two or more; unknown);
h) sex of the probable aggressor (male; female; both sexes; unknown);
i) relationship to the person receiving care [family members; partners or ex-partners; friends or acquaintances; strangers; others].

SINAN and CNES data were publicly available on the Brazilian National Health System Information Technology Department (*Departamento de Informática do SUS* - DATASUS) website.^15^,^16^ The year 2019 was selected for the study because it refers to the latest database with final data made available by the Ministry of Health, which means that the database had already undergone the cleaning process and exclusion of duplicates at the federal level. The SINAN database was accessed on November 24, 2021. The CNES database was accessed on September 20, 2021.

Incidence rates of notification of sexual violence (per 100,000 inhabitants) were calculated according to three age groups (in years: 0 to 9; 10 to 19; 20 or over), for both females and males, across the 89 health micro-regions in the state of Minas Gerais. For the numerator, we took into consideration the total number of notifications of sexual violence reported on the SINAN in 2019, according to sex, age group and health microregion;^17^ The denominator was comprised of the total population of the micro-region in 2019, according to sex and age group, based on resident population estimates.^18^ For the age group of 20 years or older, the standardized rate of notification of sexual violence was calculated using the direct method and the distribution of the Brazilian population as the standard.

The number and geographical distribution of SAP/VS were described according to the 14 health macro-regions^17^ of the state, within the four classifications of the Service.

In order to analyze the distance traveled by people with notifications of sexual violence to the nearest AIP/VS referral service and within the same health macro-region, the calculation of distance – in a straight line and in kilometers – between the municipality of notification and the nearest referral service (municipality with the AIP/VS referral service classification, within each health macro-region) was performed. Macro-regions that were not registered in the CNES for this classification were not included in this analysis, namely: Noroeste, Nordeste, Leste e Leste do Sul. The Euclidean Distance function (ArcGIS software) and meshes provided by the Institute for Applied Economic Research/Ministry of Planning and Budget (*Instituto de Pesquisa Econômica Aplicada/Ministério do Planejamento e Orçamento -* IPEA/MPO) were used to calculate the distance.^19^

For comparisons between the state’s health macro-regions, we took into account the maximum distances calculated between the place of notifications and the specialized care service, considering that this would be the longest distance traveled by a person with a notification of sexual violence to the nearest AIP/VS referral service within the macro-region.

R^20^ software and the ArcGIS software (version 10.5) were used for the analyses.

The study was based on secondary, aggregate data, without personal identification. As these data are available in public domain databases, the project was exempted from approval by a Research Ethics Committee (REC).

## RESULTS

In 2019, 4,429 notifications of sexual violence were identified in Minas Gerais; one case was classified as “unknown” sex, nine cases had “unknown” age and one case had “unidentified” municipality of residence. These 11 cases with missing data for sex, age and municipality of residence were excluded from the study, and a total 4,418 notifications remained to be analyzed: 87.0% were female, 72.0% were children and adolescents (0 to 19 years old) and 18.0% were in the age group of 20 years and older, showing “rape” as the most commonly reported type of sexual violence: 60.8% (Table 1 and Table 2).

**Table 1:** Description of the characteristics of notifications of sexual violence against children and adolescents, by sex and age group, state of Minas Gerais, Brazil, 2019

Variables	Children **(n = 1,473)**					Adolescents (n = 1,706)			
	**Female** **(n = 1,130)**		**Male** **(n = 343)**			**Female** **(n = 1,541)**		**Male** **(n = 165)**	
	**n**	**%**	**n**	**%**		**n**	**%**	**n**	**%**
Race/skin color									
White	346	30.6	108	31.5		407	26.4	47	28.5
Black	92	8.1	38	11.1		222	14.4	21	12.7
Asian	9	0.8	–	–		13	0.8	1	0.6
Mixed-race	560	49.6	151	44.0		794	51.5	84	50.9
Indigenous	6	0.5	1	0.3		7	0.5	2	1.2
Unknown	117	10.4	45	13.1		98	6.4	10	6.1
**Marital status**									
Single	–	–	–	–		1,159	75.2	108	65.5
Married or in consensual union	–	–	–	–		43	2.8	2	1.2
Separated	–	–	–	–		3	0.2	–	–
Not applicable	1,130	100.0	343	100.0		227	14.7	41	24.8
Unknown	–	–	–	–		109	7.1	14	8.5
**Disability/disorder**									
Has disability	24	2.1	25	7.2		133	8.6	29	17.6
Does not have disability	946	83.7	277	80.8		1,277	82.9	114	69.1
Unknown	160	14.2	41	12.0		131	8.5	22	13.3
**Pregnancy status**									
Yes	–	–	–	–		112	7.3	–	–
No	1,130	100.0	–	–		923	59.9	–	–
Not applicable	–	–	343	100.0		308	20.0	165	100.0
Unknown	–	–	–	–		198	12.8	–	–
**Previous occurrence of violence**									
Yes	323	28.6	110	32.1		622	40.4	60	36.4
No	382	33.8	118	34.4		679	44.0	69	41.8
Not applicable	425	37.6	115	33.5		240	15.6	36	21.8
**Place of occurrence**									
Home	740	65.4	213	62.1		933	60.6	84	50.9
Collective housing	8	0.7	1	0.3		16	1.0	7	4.3
School	63	5.6	27	7.8		21	1.4	4	2.4
Sports facility	2	0.2	1	0.3		7	0.4	2	1.2
Bar or similar establishment	3	0.2	4	1.2		16	1.0	–	–
Public thoroughfare	22	2.0	17	5.0		181	11.8	15	9.1
Trade/services	2	0.2	2	0.6		13	0.8	4	2.4
Industries/construction site	–	–	–	–		7	0.5	1	0.6
Other	98	8.7	24	7.0		160	10.4	27	16.4
Unknown	192	17.0	54	15.7		187	12.1	21	12.7
**Type of sexual violence** ^a^									
Sexual harassment	496	38.8	118	29.6		575	30.4	55	26.7
Rape	580	45.4	218	54.6		1,156	61.2	119	57.8
Child pornography	53	4.1	14	3.5		37	2.0	6	2.9
Sexual exploitation	38	2.9	20	5.0		64	3.4	9	4.4
Others	112	8.8	29	7.3		56	3.0	17	8.2
**Number of aggressors involved**									
One	812	71.8	249	72.6		1,217	79.0	105	63.6
Two or more	151	13.4	52	15.2		248	16.1	50	30.3
Unknown	167	14.8	42	12.2		76	4.9	10	6.1
**Sex of the agressor**									
Male	880	77.9	289	84.2		1,452	94.2	146	88.5
Female	58	5.1	15	4.4		23	1.5	4	2.4
Both sexes	23	2.0	4	1.2		16	1.0	5	3.0
Unknown	169	15.0	35	10.2		50	3.3	10	6.1
**Relationship to the person receiving care** ^b^									
Family members	357	34.5	93	29.0		394	26.0	30	18.6
Partners or ex-partners	–	–	–	–		151	10,0	1	0,6
Friends or acquaintances	289	27.9	107	33.3		468	30.8	60	37.3
Strangers	81	7.8	28	8.7		265	17.4	17	10.6
Others	309	29.8	93	29.0		239	15.8	53	32.9

a) Variable with multiple-choice possibility, in which, among children, 1,279 records were identified for females and 399 for males, and among adolescents, 1,888 records for females and 206 for males; b) Variable with multiple-choice possibility, in which, among children, 1,036 records were identified for females and 321 for males, and among adolescents, 1,517 records for females and 161 for males.

**Table 2:** Description of the characteristics of notifications of sexual violence among adults, by sex, state of Minas Gerais, Brazil, 2019

Variables	Adults (n = 1,239)				
	**Female (n = 1,171)**			**Male (n = 68)**	
	**n**	**%**		**n**	**%**
Race/skin color					
White	393	33.6		32	47.0
Black	186	15.9		5	7.4
Asian	12	1.0		–	–
Mixed-race	528	45.0		29	42.7
Indigenous	2	0.2		–	–
Unknown	50	4.3		2	2.9
**Mariatal status**					
Single	622	53.1		50	73.5
Married or in consensual union	284	24.2		10	14.7
Separated	125	10.7		1	1.5
Widowed	31	2.7		2	2.9
Not applicable	15	1.3		–	–
Unknown	94	8.0		5	7.4
**Disability/disorder**					
Has disability	218	18.6		29	42.6
Does not have disability	833	71.1		32	47.1
Unknown	120	10.3		7	10.3
**Pregnancy status**					
Yes	112	9.6		–	–
No	781	66.7		–	–
Not applicable	128	10.9		68	100.0
Unknown	150	12.8		–	–
**Previous occurrence of violence**					
Yes	412	35.2		18	26.5
No	616	52.6		37	54.4
Unknown	143	12.2		13	19.1
**Place of occurrence**					
Home	644	55.0		29	42.6
Collective housing	15	1.3		–	–
School	6	0.5		2	2.9
Sports facility	4	0.3		–	–
Bar or similar establishment	19	1.6		–	–
Public thoroughfare	242	20.7		21	30.9
Trade/services	24	2.0		–	–
Industries/construction site	3	0.3		1	1.5
Other	110	9.4		7	10.3
Unknown	104	8.9		8	11.8
**Type of sexual violence** ^a^					
Sexual harassment	304	23.0		13	18.0
Rape	938	71.1		54	75.0
Sexual exploitation	30	2.3		3	4.2
Others	48	3.6		2	2.8
**Number of aggressors involved**					
One	927	79.2		39	57.4
Two or more	193	16.4		26	38.2
Unknown	51	4.4		3	4.4
**Sex of the agressor**					
Male	1.124	96.0		64	94.2
Female	11	0.9		2	2.9
Both sexes	3	0.3		–	–
Unknown	33	2.8		2	2.9
**Relationship to the person receiving care** ^b^					
Family members	61	5.3		6	9.2
Partners or ex-partners	348	30.1		8	12.3
Friends or acquaintances	251	21.7		20	30.8
Strangers	392	33.9		25	38.5
Others	103	9.0		6	9.2

a) Variable with multiple-choice possibility, in which 1,320 records were identified for females and 72 for males; b) Variable with multiple-choice possibility, in which 1,155 records were identified for females and 65 for males.

Regarding all age groups, females showed a higher frequency of notifications of sexual violence, exceeding 90% among people aged 10 years and older. The majority of notifications were related to mixed-race/Black people, of both sexes, and in all age groups. Sexual violence against people with disabilities was more frequently reported in males, for all age groups, reaching 42.6% of adults. Among female adolescents and adults, previous occurrences of sexual violence were reported in 40.4% and 35.2% of the cases, respectively. Regardless of sex and age group, the primary place of occurrence was the residence, and the most commonly reported type of sexual violence was rape (Table 1 and Table 2).

With regard to females, aged 0 to 9 and 10 to 19 years old, the main aggressors were family members (34.5% and 26.0%, respectively) and friends or acquaintances (27.9% and 30.8%, respectively); among adult females, the main aggressors were strangers (33.9%) and partners/ex- partners (30.1%) (Table 1 and Table 2).

Among males, in the age groups of 0 to 9 years and 10 to 19 years, the main aggressors were friends or acquaintances (33.3% and 37.3%, respectively), followed by family members (29.0% and 18.6%, respectively); among adult males, the main aggressors were strangers (38.5%) and friends or acquaintances (30.8%) (Table 1 and Table 2).

**Figure 1:**
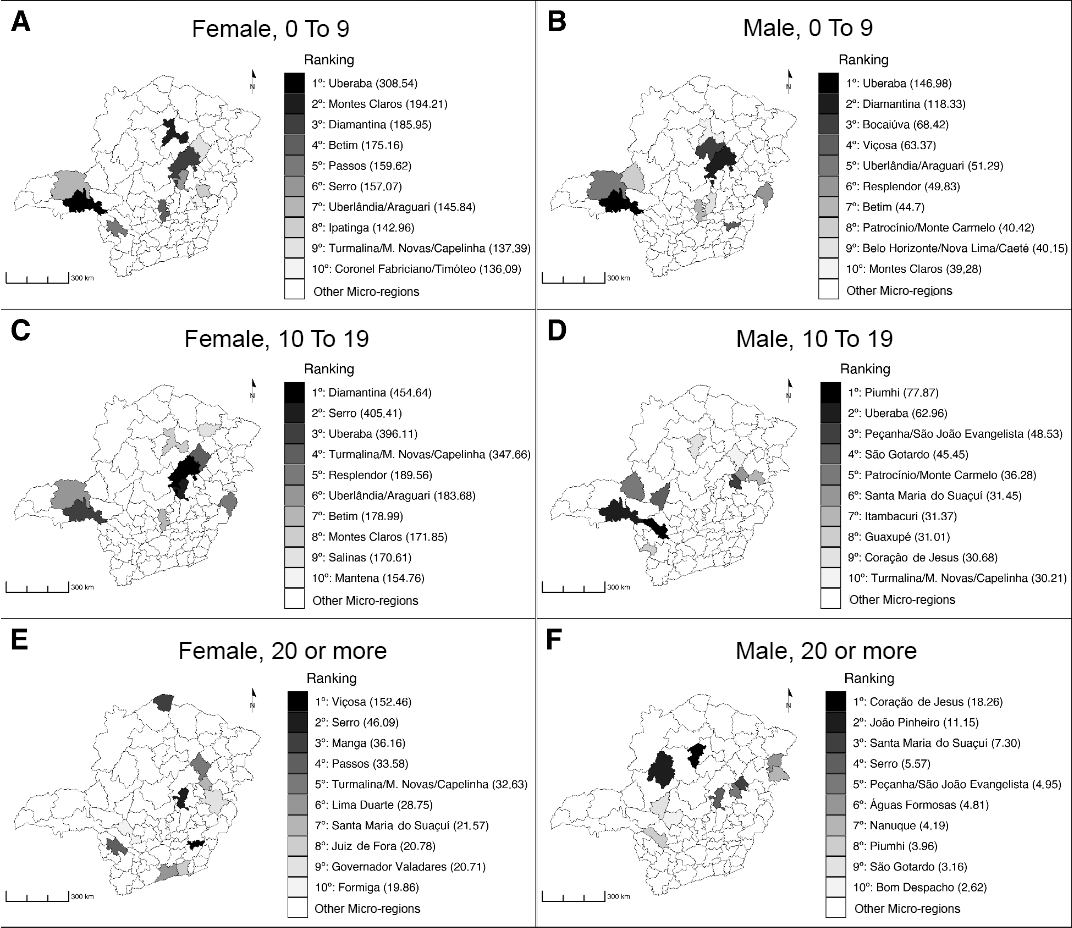
Ranking of the ten health micro-regions with the highest incidence rates of violence notification, by sex and age groups, state of Minas Gerais, Brazil, 2019

The highest incidence rates of notification were found among female adolescents: 455/100,000 inhabitants. in Diamantina; 405/100,000 inhabitants in Serro; and 396/100,000 inhabitants in Uberaba (Figure 1 and Supplementary Figure).

In the state of Minas Gerais, in July 2019, the AIP/VS, AAP/VS and CV/VS referral services were the most frequently registered in the CNES, with 28, 20 and 19 services respectively (Table 3). Among the 89 health microregions in the state, only 30 had some type of SAP/VS registered in the CNES (Table 3). Regarding the 14 health macro-regions, four of them (Leste; Leste do Sul; Nordeste; Noroeste) did not have AIP/VS services, and in the Southeast microregion no SAP/VS was identified. It is noteworthy that only seven referral services for termination of pregnancy (*interrupção da gravidez* - GI) were registered in the whole state. The Centro macro-region showed the highest number and of registered services (Table 3).

**Table 3:** Distribution of the four classifications of Care Service for People Subjected to Sexual Violence within the Brazilian National Health System, registered in the National Health Establishment Registry, state of Minas Gerais, Brazil, July 2019

Health micro-region	Health macro-region	Comprehensive care	Outpatient care	Termination of pregnancy	**Evidence collection**
**Almenara/Jacinto**	Nordeste	–	1	–	–
**Araxá**	Triângulo do Sul	1	–	–	–
**Belo Horizonte/Nova Lima/Caeté**	Centro	4	–	4	6
**Betim**	Centro	2	–	–	2
**Congonhas**	Centro-Sul	–	1	–	–
**Conselheiro Lafaiete**	Centro-Sul	1	-	–	1
**Contagem**	Centro	3	2	–	2
**Coração de Jesus**	Norte	–	–	–	1
**Diamantina**	Jequitinhonha	1	–	–	–
**Divinópolis**	Oeste	1	–	–	1
**Governador Valadares**	Leste	–	1	–	–
**Ipatinga**	Vale do Aço	1	1	–	–
**Itabira**	Centro	1	–	1	1
**Itajubá**	Sul	1	–	–	1
**Itaobim**	Nordeste	–	1	–	–
**João Monlevade**	Centro	1	1	–	–
**Juiz de Fora**	Sudeste	1	1	–	1
**Lima Duarte**	Sudeste	–	3	–	–
**Montes Claros**	Norte	1	–	–	1
**Passos**	Sul	1	–	–	–
**Patrocínio/Monte Carmelo**	Triângulo do Norte	–	2	–	–
**Pedra Azul**	Nordeste	–	2	–	–
**Pouso Alegre**	Sul	1	–	–	–
**São João Del Rei**	Centro-Sul	–	1	1	1
**São João Nepomuceno/Bicas**	Sudeste	–	1	-	-
**São Sebastião do Paraíso**	Sul	1	–	–	–
**Três Corações**	Sul	1	–	–	–
**Uberaba**	Triângulo do Sul	3	–	–	–
**Uberlândia/Araguari**	Triângulo do Norte	2	1	1	1
**Unaí**	Noroeste	–	1	–	–
**Total**		**28**	**20**	**7**	**19**

**Figure 2:**
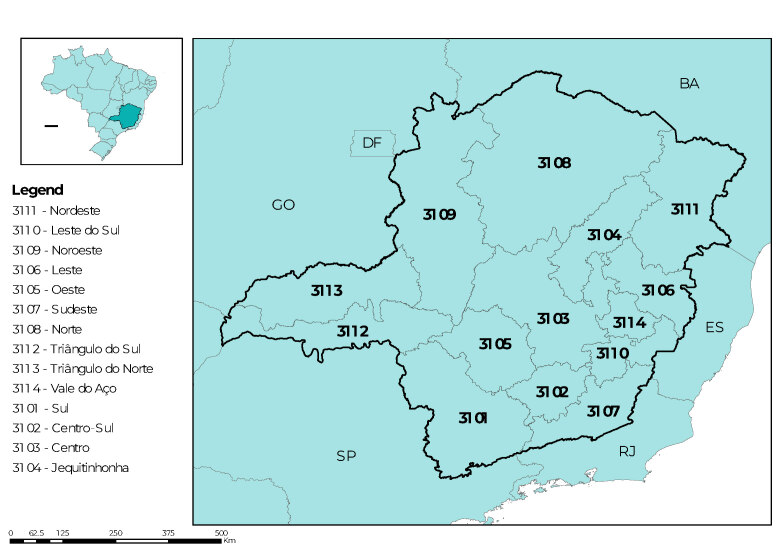

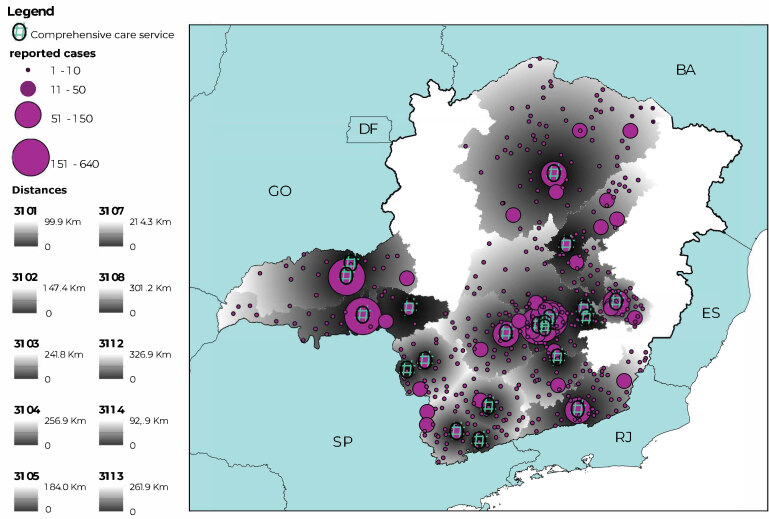
Distance between municipalities with notifications of cases of sexual violence and municipalities that offer services registered in the National Health Establishment Registry classified as Comprehensive Care for People Subjected to Sexual Violence, according to health macro-regions, state of Minas Gerais, Brazil, 2019

As for the distance traveled by a person with a notification of sexual violence to the nearest AIP/VS referral service within the health macro-region, maximum distances ranging from 327 km to 93 km were observed. The five macro-regions with the largest maximum distances were: Triângulo do Sul (327 km); Norte (301 km); Triângulo do Norte (262 km); Jequitinhonha (257 km); and Centro (242 km). Average distances ranged from 42 km (Vale do Aço) to 149 km (Norte) (Figure 2).

## DISCUSSION

Notifications of sexual violence in 2019, in the state of Minas Gerais, showed a worrying scenario: over 12 notifications per day in the state’s health services. The records were concentrated in females, children and adolescents and mixed-race/Black people, with the house being the primary place of violence perpetration and males the main aggressors.

The evaluation of the distribution of the SAP/VS showed care gaps in the provision of AIP/VS in four health macro-regions; the absence of any type of SAP/VS in one (1) macro-region of the state stood out. Regarding the distances traveled by people with notifications of sexual violence, among the ten macro-regions with a referral service for AIP/VS, five had an average distance greater than 100 km, a considerable displacement for people subjected to sexual violence. In six macro-regions, the maximum distance traveled to a referral service for AIP/VS was even greater, exceeding 200 km. Overall, these findings are similar to those of other studies on gender-based violence and sexual violence, reinforcing that mixed-race/Black, young women and sometimes with disabilities are more vulnerable in this regard.^14^,^21^ These findings show how the intersection of the dimensions of gender, race/skin color, age and disability is related to the experience of sexual violence, reflecting the inequalities imposed by distinct and overlapping power relations that affect people in different ways.^22^

Despite the existing legal coercion mechanisms, such as the Maria da Penha Law (Law No. 11,340, of August 7, 2006)^23^ and the Femicide Law (Law No. 13,104, of March 9, 2015),^24^ in addition to public initiatives to address and provide care for people who experienced sexual violence, the historical prevalence of this health condition among women and its chronicity, often associated with these occurrences, demonstrate that society and especially women are still entangled in mental models and/or imagery, produced under power relations and domination based on a symbolic order, whose main beneficiaries are men and their intentions.^25^

Federative pacts, such as the National Plan to Confront Sexual Violence against Children and Adolescents,^26^ aiming at ensuring protection for children and adolescents, have not prevented the highest occurrence of this form of violence against these age groups, showing a historical tradition of intrafamilial abuse, as supported by some findings of the present study, such as the identification of the residence as the primary place of the aggression; and predominantly committed by family members, whether the person subjected to violence is female or male.

Poor access to protection policies and reporting mechanism are factors directly related to the worsening and emergence of new cases of sexual violence against children and adolescents, as demonstrated here; and especially against girls, whose discursive place occupied by their bodies still includes the sadistic idea of property and dominance, sustained by a colonized patriarchal system. In these cases, early interventions performed by health professionals, have been an important strategy, pointed out by research in various fields of knowledge interested in the subject, such as psychology.^27^

Data from SINAN reveal a partial picture of this serious problem: a significant underreporting of cases of sexual violence. Although it is mandatory to report this form of violence against people of any age or sex, this record depends on the academic training of health professionals. The notification of sexual violence also depends on the understanding and sensitization of these professionals in the healthcare setting, in the guidance on care and in the promotion of the health of the affected individuals, in addition to the correct completion of the Notification Form as an instrument for recording essential data for the epidemiology of the health condition and the public health response actions.^14^

In this sense, it is necessary to consider the following hypotheses for approaching the issue: difficulty in understanding and sensitivity of professionals regarding the notification and the care for children and adolescents victims of this violence and its prevention; difficulty in identifying and recording sexual violence in adult males; difficulty in understanding and recording cases in women when the aggressors are their intimate partners or friends/acquaintances; and a higher registration of more severe cases that seek care in specialized centers.

Even considering the existence of underreporting of sexual violence, the incidence rates based on the notification of cases proved to be alarming. In situations where not all cases that receive care are reported, the calculation of the incidence rate may unfairly highlight microregions with municipalities that are more sensitized and active in the surveillance of sexual violence as having the highest rates.

The assessment of the distribution of the SAP/VS showed care gaps or points of low resolubility in the following macro-regions: Noroeste, Leste do Sul, Leste and Nordeste. Health macro-regions constitute the territorial basis for planning tertiary health care, offering hospital health services with higher-density technology. Health micro-regions, on the other hand, are the territorial basis for planning secondary health care, by providing outpatient and hospital services of medium complexity and, exceptionally, some high complexity services. In summary, the macro-region encompasses all three levels of tertiary, secondary and primary health care, while the micro-region encompasses services of primary and secondary health care.

In this context, the territorial unit of the macro-region should have registered health facilities of the three levels of health care, to assist people subjected to sexual violence, as well as the unit of the micro-region should have registered existing outpatient services within its jurisdiction.

A study based on data from notifications of sexual violence in Minas Gerais, including specialized services in the state, observed that in 2017, there were 40 health centers registered as SAP/VS, distributed in 11 out of the 14 macro-regions in the state, with the majority (37.5%) located in the Centro macro-region. The same study showed that approximately 10% of the cases of rape among women in the state, that same year, occurred in health macro-regions where there were no specialized healthcare centers and 23% occurred in macro-regions with only one specialized healthcare center.^14^ This study identified 48 healthcare centers registered as SAP/VS, that is, only eight new services were registered in the two-year period. Moreover, Leste do Sul macro-region, where significant rates of sexual violence were found, the care gap remains. These findings reveal that despite the investment made by the Brazilian government in the organization of SAP/VS – especially since 2015, when the Ministry of Health created mechanisms for registration and remuneration of healthcare centers providing this type of care – there has not been significant expansion of these services in Minas Gerais.

In addition to the care gaps found, the distances that people subjected to sexual violence must travel to have access to comprehensive care services hinder timely care, compromising the provision of care within 72 hours after the act of sexual violence, as recommended by health institutions.

This study had some limitations: (i) the calculation of rates by health microregion could hide particular situations in specific municipalities; (ii) the distances calculated corresponded only to the territory of the macro-region where the case occurred, while in the cases that occurred in border regions, there is the possibility of receiving care in other health macro-regions in Minas Gerais and even in other states, although it was not possible to consider these situations in this study, given that the distances were calculated in a straight line and under different aspects, both related to the organization of the road network and of cultural nature, imposing greater distances to be traveled by people seeking care; and finally (iii) services providing care to people subjected to sexual violence may not be registered with the CNES.

As a potential strength of the study, it can be seen that, despite the underreporting, the rates of sexual violence are much higher than the rates of several diseases considered as public health emergencies. Therefore, addressing sexual violence in the state of Minas Gerais requires policies that better integrate the training of healthcare professionals, and a coordinated service network available throughout the territory, as recommended by the Regionalization Master Plan.^17^

It can be concluded that the study presented a picture of sexual violence in Minas Gerais in 2019, characterized by the concentration of cases in females, children and adolescents, mixed-race/Black people, and its occurrence in the domestic, intimate and family space. If, on the one hand, high incidence rates of notification were found in the health micro-regions of the state, on the other hand, a scarcity of care services for people subjected to sexual violence (*Serviço de Atenção às Pessoas em Situação de Violência Sexual* - SAP/VS) and care gaps between these micro-regions, were identified, especially services classified as Outpatient Care for People Subjected to Sexual Violence (*Atenção Ambulatorial às Pessoas em Situação de Violência Sexual* - AAP/VS). The maximum distances that people with notifications of sexual violence must travel to receive care in a Comprehensive Care for People Subjected to Sexual Violence” (*Atenção Integral às Pessoas em Situação de Violência Sexual* - AIP/VS) service -, are high, with average distances exceeding 100 km. These results highlight the need for improving public policies and their implementation, strategic planning in the location of services and expansion of specialized care coverage for cases of sexual violence in the state of Minas Gerais.
